# Supplementary material for: Factors affecting utilization of mental health services from Primary Health Care (PHC) facilities of western hilly district of Nepal
Source: PLoS One. 2021 Apr 30;16(4):e0250694. doi: 10.1371/journal.pone.0250694 (PMC8087454; doi:10.1371/journal.pone.0250694)
Supplement: S1 Table — (DOCX) [file pone.0250694.s001.docx]

| **Municipality** | **PHCCs** | **HPs** | **UHCs** | **CHUs** | **Total** |
| --- | --- | --- | --- | --- | --- |
| Malarani Rural Municipality |  | 6 |  | 2 | 8 |
| Panini Rural Municipality |  | 8 |  | 1 | 9 |
| Chhatradev Rural Municipality | 1 | 6 |  |  | 7 |
| Sandhikharka Municipality |  | 6 | 1 |  | 7 |
| Sitganga Municipality | 1 | 6 |  |  | 7 |
| Bhumekasthan Municipality |  | 7 |  | 1 | 8 |
| Total | 2 | 39 | 1 | 4 | 46 |

S1 Table: Primary Health Care facilities in Arghakhanchi district
