# Supplementary material for: Factors affecting utilization of mental health services from Primary Health Care (PHC) facilities of western hilly district of Nepal
Source: PLoS One. 2021 Apr 30;16(4):e0250694. doi: 10.1371/journal.pone.0250694 (PMC8087454; doi:10.1371/journal.pone.0250694)
Supplement: S1 Appendix — (DOCX) [file pone.0250694.s002.docx]

## Interview Guidelines for Nearest Caretaker/Patients

| **Topics and Issues** | **Main Question** | **Follow up questions** | **Probe** |
| --- | --- | --- | --- |
| Ice-breaking | 1. Could you please provide your brief introduction? | 1. Before you/your relative getting mentally ill, what was your thinking regarding mental illness? 2. How does society perceive mental illness? 3. What do you think caused the mental illness? |  |
| Service Utilization | 1. Can you please say about all your experiences from starting of the symptoms of this disease until now? | 1. Where did you visit for the diagnosis? Why? 2. Where did you visit for treatment? Why? 3. What were the factors that affected you seeking mental health care? 4. What were the factors that affected you visiting PHC for mental health care? 5. What were the factors that affected you getting mental health care from PHC? | What were the factors affecting mental health service utilization at:  Individual level  Interpersonal level  Organizational level  Community level  Policy level |
| Recommendations | 1. What do you think should be done to address those factors hindering utilization and delivery of mental health services at PHC? | | |

## Interview Guidelines for Health Care Service Providers

| **Topics and Issues** | **Main Questions** | **Follow up questions** | **Probe** |
| --- | --- | --- | --- |
| Ice-breaking | 1. Could you please provide your brief introduction? 2. What are the major services provided by this facility? | Is the delivery of health services by this facility of good quality? Why?  And what about mental health services? |  |
| Service Utilization | 1. Do people from this community visit this facility for seeking mental health services? Why, why not? | 1. What factors did you identify supporting people in this community to utilize mental health care? 2. What factors did you identify hindering people in this community to utilize mental health care? | What are the factors affecting mental health service utilization at:  Individual level  Interpersonal level  Organizational level  Community level  Policy level |
| Recommendations | 1. What are your recommendations for improving mental health care utilization at this health facility? | | |

## Interview Guidelines for Health Administrators/Elected Representatives

| **Topics and Issues** | **Main Questions** | **Follow up questions** | **Probe** |
| --- | --- | --- | --- |
| Ice-breaking | 1. Could you please provide your brief introduction? 2. What do you think about the general health situation of this district? 3. What are the major services provided by primary health care settings of this district? | 1. Is the delivery of health services by the primary health care settings of good quality? Why, why not? 2. And what about mental health services? |  |
| Service Utilization | 1. What are the factors that support utilization of mental health services by community people in primary health care facilities in this district? 2. What are the factors that hinder utilization of mental health services by community people in primary health care facilities in this district? | | What are the factors affecting mental health service utilization at:  Individual level  Interpersonal level  Organizational level  Community level  Policy level |
| Recommendations | 1. What are your recommendations for improving mental health care utilization from primary health care settings of this district? | | |
