# Supplementary material for: Factors affecting utilization of mental health services from Primary Health Care (PHC) facilities of western hilly district of Nepal
Source: PLoS One. 2021 Apr 30;16(4):e0250694. doi: 10.1371/journal.pone.0250694 (PMC8087454; doi:10.1371/journal.pone.0250694)
Supplement: S1 Transcript — (DOCX) [file pone.0250694.s005.docx]

Interviewer (I)- Namaskar

Participant (P) – Namaskar

I: My name is Gaurav Devkota. I am currently studying Masters in Public health at Patan Academy of Health Sciences. I am requesting you to participate in the research titled factors affecting utilization and delivery of mental health services at primary health care centers of Argakhanchi district. Are you willing to participate in this research?

P: Yes I am

I: First of all please give me your brief introduction

P: I work in the health section of the gaunpalika. I am the health coordinator.

I: In your opinion what is the basic health condition of this gaunpalika?

P: The basic health condition of this gaunpalika, the programs as being directed and on conditional grant by the Nepal government is being delivered through health facilities. After the introduction of local government, it is being problematic for us to explain in local government as there have been some problems in the understanding of the technical part. However, in terms of delivering health services, the services are okay, they are no less and there have been no complaints from the public.

I: What are the major health services being provided from the primary health care centres of this municipality?

P: The services being provided from the primary level health centres of this municipality are mainly nutrition program, ARI control program, Diarrhea control program and CB IMNCI program under child health program. Another is safe motherhood program. Under this Antenatal check up, Post natal check up, safe delivery services in birthing centers. Similarly, Family planning program, FCHV program, TB and Leprosy control program, HIV and STD control program, School health education program, and curative services are being provided from the health facilities of this rural municipality.

I: How is the quality of the health services being provided from the health facilities?

P: Talking about the quality, basically the quality is measured by the central quality assurance body. We have also felt that they could have assessed the quality. On the basis of resources, human resources, availability of resources, the health services being provided can be considered to be of fair quality in Nepal in the present context. Referring to the quality of materials and drugs we do not have the capacity to measure the quality and also do not have the responsibility. Nevertheless the services we deliver and the medicines that we distribute have been proving effective, we have not heard any negative things about it, so we are content about it.

I: What is the status of mental health services being provided by the health facilities of this municipality?

P: Along with the above mentioned services, mental health related services is also provided from the primary health care facilities of this municipality, however, there is no separate package, no trained health worker specially for mental health. But we at our level with the available health worker on the basis of their experiences and some knowledge provide counseling to the patient with mental health problem, try to figure out the status of the patient along with his family status, history taking on what triggered him to have mental health problem. Based on these, health workers try to find out the problem and counsel them accordingly. Talking about the curative services, we do not have doctors at our level. Given that we do not have the mental health specialist doctors it cannot not be said that the treatment of the mental health is success. As for counseling the health workers are providing them and it is good.

I: What are the facilitators for utilizing the mental health services by the people in this municipality?

P: For utilization of mental health services in this municipality, at times we have informed all the health workers in the facility, FCHV, people in health mother’s group about mental health that if there is any individual with the mental health problem, we have asked them to inform the family member and in absence of the family member inform his relatives and send him to the health facility. In case of any patient we have asked them to inform us and in this way we have made easy environment to reach to health facilities.

I: I want to repeat the question again. How are service utilizers being supported at intrapersonal, interpersonal, institutional level you have already spoken about it, community level and policy level to utilize the health services?

P: At policy level, people utilizing health service, health workers giving health education also comes under the policy level. Field visit by the health workers, counseling about their mental health problems during their visits at the health facility also motivate them and so they keep coming to take the health services in the municipality.

I: Similarly, what are the barriers for utilization of mental health services?

P: There are service utilizers who come to seek mental health services. But at the present context we cannot tell that we have the manpower that is capable of providing the health services to fully treat the mental health patient according to their health need and satisfy them. We do not have the mental health specialist doctors, so this has been one of the barriers. So if training could be provided to the health workers on mental health, the counseling could have been more effective. Also the knowledge about the referral would be added. Unavailability of training to the health worker about the mental health is being one of the barriers. So, if there is a special training package of mental health, then we could have reduced the problem to some extent. And unavailability of trained specialist doctors, in absence of them we cannot prescribe the medicines related to mental health. In absence of the mental health specialist doctors we have been forced to limit our mental health services to counseling only.

I: The things that you have shared are about the barriers for providing services but what could be the facilitators and barriers for service utilizers to utilize the mental health services?

P: For service utilizers if they or their family members could be assured that if we go to the health facility our mental health problem will be solved, then they would come to utilize the service. But if they only receive counseling and do not get treatment for their health problem then they would search for other alternatives, go to higher level health facilities. In primary health centers if their health problem is in initial stage they come for the counseling services, and once they know that they will not receive curative health services for mental illnesses, they and their parents will not come to the health facility and go to higher level health facility where they will receive the treatment. So, until and unless the treatment system is not made available, there will be barrier in the utilization.

I: Likewise are there any barriers at their intrapersonal level and community level?

P: At their intrapersonal level, in our society, the perception towards the mental health patient is slightly different from the perception towards normal person. His brain does not function well, he is a mentally ill person, one cannot talk more to him, and people do not get more close to him. Only the family is little close to him due to the family bond but other neighbors, community people who should be close does not try to get close to him and so there is the barrier for the patient to get the service on time. Additionally at family level also, there are many other problems, one has to earn money. It is mostly the family member and the close acquaintances that help the patient to utilize the mental health service. In community also, after suffering from the mental health problem the perception towards him changes. And people even do not want to listen to him and says that his brains are little weak, why to waste time by listening to what he is saying we have to go to our work. We feel the patient is neglected and he does not receive the love from family, neighbors, people in the community which is a problem. But if there is a plan and policy of Nepal government to develop manpower who can conduct awareness program in the community regarding mental health then condition of such patient would not be more worse and they themselves in initial stage could come to seek counseling services and for treatment also where they should go they would have received the information. So it would have been good if the training related to mental health could be made available to all the health workers promptly.

I: What are the facilitators and barriers in providing mental health services from the health facilities?

P: About providing mental health services, basically the community people who are unwell come to the health facility. Some have some problem and the others have next problem, we can sense sympathy to each other. They sympathize the patient with mental health problem; they show some pity and talk about what could be the problem and how they can be treated. We get support from other service utilizers as well. From the service provider side also we try to maintain their privacy while providing services. His confidentiality is maintained by taking him to the separate room and assess his stress and tension. This has helped us in exploring his problems and conveying him the right suggestion. Now talking about the barriers, if we could only avail medicines, equipments, booklets, brochures, many people who are literate could gain some knowledge. In absence of many booklets related to mental health and unavailability of medicines and specialist doctors to prescribe those medicines, these could be considered as the barriers at this moment.

I: At the end, what are your recommendations for improving mental health service utilization and delivery at primary health care centers of this municipality?

P: Mental health problems are the unimaginable health problems in the life of the people. It is because not the physical health problem but mental health problem acts as the barrier in the upcoming life of the people. Once he suffers from mental health problem he cannot think about this and that, cultures, traditions, and upcoming future. So, to abrogate this problem, if possible there should be the establishment of separate mental health counseling centre in the primary health care centres by adding manpower. At present the health facilities under construction are separating a room with privacy, so there may not be the problem for confidential room now. With the available health workers it would be difficult to manage the mental health as the counseling process takes long time and there are other activities also to be performed by the health workers. At present we have to be satisfied with all the services that are being provided. Hence if there is a health worker specially trained for the mental health that can counsel and conduct awareness programs in the community, then only the services provided would be effective and also the service utilizers would feel as if they got good services and their relatives would be satisfied too. So it would be better to keep such programs.

I: Finally, Please share if there is anything that I forgot to ask and anything you would like to add regarding the research.

P: The topic that you have come for the research is very important topic. At present when there is no separate program launched, you have come here as a part of your research to know about the general situation, service utilization situation, facilitators and barriers for service utilization and delivery and it is highly commendable. In upcoming days also, if you could do such visit, then we would be able to share the services being provided by our health facilities and health situation of the people. This would aid in the policy development. Also many NGO and INGO are working in the country and they are conducting various health programs but might be I am unaware of but there is no mental health focused NGO/INGO. At least our Argakhanchi district has not been able to sense it. If any NGO/INGO comes, then Nepal government could give them permission and suggestion to work in the sector of mental health, we feel that it would have been good. Also, the body responsible for the awareness program in health sector should specially prioritize mental health at the community level. To conduct this program at the community level, Nepal government should manage some conditional budget so that we can hire the manpower to conduct awareness program, it would have been effective and the number of people with mental health problems would have been less and these patients would have been managed on time. Hopefully, in the upcoming years mental health programs will be included by the Nepal government, this year no budget is mentioned for such programs, we have seen the budget ceiling. And if this could be done it would be better.

I: At the end I would like to thank you for giving time for this interview and research despite your busy schedule.

P: Thank you to you too for giving us the opportunity to share about the services provided by the health facilities of this municipality particularly mental health services. No one has come for doing research about it, I am so glad that you came, and to be able to share with you about the situation I feel privileged. I am hopeful that in the upcoming days also you will visit us and share about the findings of the research and if Nepal government or any other organizations are willing to launch program on this regard. With this I would also like to give you a special thank you.

I: Thank you.

P: Thank you.
