# Supplementary material for: Factors affecting utilization of mental health services from Primary Health Care (PHC) facilities of western hilly district of Nepal
Source: PLoS One. 2021 Apr 30;16(4):e0250694. doi: 10.1371/journal.pone.0250694 (PMC8087454; doi:10.1371/journal.pone.0250694)
Supplement: S2 Transcript — (DOCX) [file pone.0250694.s006.docx]

Interviewer: Namaskar.

Participant: Namaskar.

I: My name is Gaurav Devkota. I am currently studying Masters in Public health at Patan Academy of Health Sciences. I am requesting you to participate in the research titled factors affecting utilization and delivery of mental health services at primary health care centers of Argakhanchi district. Are you willing to participate in this research?

P: Yes, I am.

I: Please provide your brief introduction.

P: My name is BA and I am chief of Malarani Rural Municipality. I have also been chief of Khana VDC for two tenures. I am also the chief of Jilla Sahakari Sangh and Gharelu tatha Sana Udhyog Mahasangh. I have been working continuously for …(disturbances in recording) from 2027 BS.

I: What do you think about the general health situation of this rural municipality?

P: I think that the general health situation of this rural municipality is not satisfactory. This rural municipality was formed by uniting 7 different VDCs and there is lack of health posts in different places. We just started birthing centres at 4 places, but for the 9 wards in this rural municipality (birds chirping) there is requirement of 9 Health Posts and we also have a vision of a rural municipal level hospital. And as per the policy of government of Nepal there should have been a 15 bedded hospital which is not present in this rural municipality as well as there is no placement/posting of MBBS doctors, so the general health situation of this rural municipality is worse and health service users of this rural municipality have no proper access to minimum basic health services.

I: What are the major health services provided by primary health care facilities of this rural municipality?

P: For zero home delivery we have been providing services through birthing centers, we have achieved full immunization status and we have also recently started health care of elderly above 84 years age at their houses every 15 days via in-charge of different health posts.

I: Are the health services provided by primary health care facilities of this rural municipality of good quality?

P: We have provided effective health services through whatever resources we possess. We have provided good management thus there has never been shortage of medicines in the health facilities and there is continuous service delivery.

I: What are the factors that support people of this community to utilize mental health services from primary health care facilities?

P: There are no health facilities providing mental health services within this rural municipality. We have forwarded proposals and are continuously lobbying to provide treatment for patients with cardiac diseases and mental illnesses within this rural municipality but at present there is no provision of treatment of mental illness within this rural municipality.

I: What factors supports mentally ill patients to utilize mental health care services from primary health care facilities?

P: It would have been better if mental health services were provided within the rural municipality but at present people with mental illnesses have to travel far for treatment. When travelling to far places it is difficult to leave mentally ill patients alone, there is much increase in expenses if the patients are also taken and there is lot of burden to manage household properly. Also, we have not been able to provide mental health services within this rural municipality. People who are capable of spending money can provide their mentally ill family members with proper treatment and medicines from different health facilities but those who are not capable of spending money face difficulties in utilizing mental health services.

I: You talked about barriers to utilize mental health services, what are the factors that support mentally ill patients to utilize mental health services from primary health care settings like Health Posts, PHCC?

P: If required, this rural municipality is always ready to provide any kind of support. There is availability of services to transport patients to district hospital or to nearby health posts. If any mentally ill patient or their family members appeals for support from this rural municipality then we provide them with money for transportation and other support through a committee.

I: At their individual level, what factors support or hinder service utilization by mentally ill patients?

P: In this rural municipality there is somewhat commercialization and here are many uneducated persons who used to leave their mentally ill family members calling them crazy “baula” but after we were elected we encouraged people that we would provide help and support if they utilize mental health services as well as conducted awareness campaigns. Though we have not provided with training but we have visited households and provided awareness regarding mental health by saying that every people has 50 to 55% mental illness in them and have taken some form of medications, so it is not good and may affect you in future if you leave mentally ill people calling them “baula” and thus should provide treatment of mental illness. Among those, maximum have taken medicines of which some go for treatment openly and some in hidden manner.

I: At institutional level, what factors support or hinder service utilization by mentally ill patients?

P: Educated people provide support for health care service utilization. For example, there are mentally ill children in schools so the teachers there who have knowledge about the disease provide support by creating awareness within school as well as in their locality and neighborhood regarding treatment of such diseases with medications and checkups which has provided positive impact in the society. And in uneducated people how hard we may try to create awareness they do not understand about the long-term medication required for curing mental illness and deny treatment or stop long term medications. The proportion of such people has been minimized after we were elected as we had asked health care service providers and teachers in different meetings and seminars to educate people and create awareness regarding mental health and its treatment.

I: What do you think are the factors that support or hinder mental health service delivery from primary health care facilities?

P: We have been providing every possible services and medicines from health facilities, positive health services are provided from the health facilities and everyone is able to utilize health services. And we have also supported mental health service delivery by providing required medicines and help if any mental illness patients are prescribed with some medications from health facilities. So, till now there is no one who has been devoid of mental health services.

I: Are there any factors at individual level of health care service providers or interpersonal level or community level or institutional level or policy level that have supported or hindered mental health care service delivery at primary health care facilities?

P: We have been providing mental health services through available human resources for health which has always been positive health services. There are few numbers of health care service providers so that there are issues of increased waiting time or no prompt treatment but there are no other shortages except for a smaller number of health care service providers. We have always asked for coordination while demanding for medicines for different diseases on the basis of the disease prevalence and have been providing medicines for different cardiac diseases, mental illnesses and “dam”. So, there is provision of health services to all, even mentally ill patients but the problem is that there are very few numbers of health care service providers and thus access to every aspect of health is not possible.

I: At policy level is there any support being provided to health care service providers to deliver mental health services?

P: We regularly organize meetings with health care service providers where we ask about different barriers they have to face during service provision as well as ask them to provide their best service to the patient. In those meetings they have stated no barriers or difficulties in delivering health services from their respective health facilities.

I: Have health care service providers stated any barriers in health service delivery during those meetings?

P: We have provided services as per our capability and to provide higher level of services we require MBBS doctors, specialist doctors and mental disease specialist. We have also been conducting health camps by bringing mental disease specialist in order to provide mental health services to severely ill patients or who cannot afford treatment. It would have been a lot easier to provide mental health services if there was availability of MBBS doctors and mental disease specialist within rural municipality but as we do not have such health care service providers thus, we have been providing mental health services by conducting camps at different times.

I: What improvements do you recommend in order for proper utilization and delivery of mental health services from primary health care facilities of this rural municipality?

P: Truly speaking, the main requirement is manpower that is doctor, we can manage budget for different medicines that are required for treatment of mental illnesses but if there are no any doctors then the medicines cannot be prescribed and we also need expert in case of any side effects from the medicines. So, the main requirement here is specialist doctor providing 24 hours service which the municipality cannot afford on its own budget so we have tried to provide high level health services by conducting health camps and bringing specialists and experts during those camps to provide mental health services. These services are provided only in 6 months to 8 months, that is when the camps are conducted and due to lack of manpower, specialist doctors we have not been able to provide mental health services on day to day basis.

I: Finally, if there were anything I missed to ask or you wish to add regarding this research, you may please add.

P: Maximum things have already been included in the interview. We have been trying hard to get mental health specialist to provide service in this rural municipality because there are low number of mental health specialist thus if we could identify interested specialist and their requirements, we could call them for different camps we conduct to provide mental health services to the population.

I: Thank you a lot for providing time for this interview despite of your busy schedule.

P: Thank you as well for providing me with the opportunity to put forward our problems and help create environment favoring mentally ill people if the problems in the community gets identified. Thank you.

I: Thank you.
