# Supplementary material for: Factors affecting utilization of mental health services from Primary Health Care (PHC) facilities of western hilly district of Nepal
Source: PLoS One. 2021 Apr 30;16(4):e0250694. doi: 10.1371/journal.pone.0250694 (PMC8087454; doi:10.1371/journal.pone.0250694)
Supplement: S3 Transcript — (DOCX) [file pone.0250694.s007.docx]

Interviewer: Let's start interview, please introduce yourself shortly.

Participant: I am working here in this Thada PHCC since 4/5 month as a medical officer

I: What are the services provided from this PHCC?

P: The main services available in this PHCC are OPD from 10 o' clock to 3 o' clock, Due to setting this time frame also the patients are treated according to the OPD basis, then there are emergency services, birthing centre is also available here where the delivery is conducted and if unable to conduct than referred. Other than these are DOTS services, and the disability friendly types of the services is also available. Another is we have our own pharmacy services available. Then as per our level there is a lab investigation service available. Additionally there is also inpatients service available. Likewise, we provide the services as much possible from our level.

I: And how is the quality of the services provided from this institution?

P: We can measure the quality of the services by taking the compliance from the service users or from their opinion and suggestion. And according to it and patients flow pattern, I think the quality provided from this facility can be considered as of good…. it is obvious that there would be some mistakes while doing some works; otherwise I think the satisfaction of the patients have been maintained.

I: What is the condition of the mental health services provided from this institution?

P: Hmmm….. We do nothing particularly focused to the mental health services. Like in the higher level institutions, the provision of the psychiatry and the counseling services are not available. However from our staff level, what we can do like, if any patient come then after performing all physical examination, if the patient have mental health problem then according to the need as counseling or medicines, the counseling will be provided from my level and from other staff level. If the patients require the medicine then the medicine which the government have been provided at free of cost will be provided. These all are services provide from our level as much as we can. The counseling services, pharmacy (pharmacology), medicine services will be provided, if it doesn’t work then we counsel them to go or refer where there is well equipped health facility.Also we counsel them to remove the negative superstitious belief they have and encourage to move where there is good place for their treatment.

I: What are the barriers or the facilitating factors for the community people to seek the mental health services service from this institution?

P: While talking about the barrier, the patients themselves are a barrier. They don’t share about their mental illness to their family and society. They don’t know about the illness or different insight comes which is different things but even if they know about illness then also they don’t want to share about their illness and hide their mental health problem which creates more problem. Also looking to the context of our community, they seek more to the Dhami, Jhakri,etc which is problematic . It have been seen that due to the believe as *Bhoot lagyo, aatma chadyo,* numerous example have been seen in the community who seeks Dhami Jhakri for care. We have tried to reduce such practices from our level, however as the belief evolves from the very beginning, it is very hard to reduce 100% then also somewhat reduction have been seen. It is one barrier.

The another barrier is …………………… rather than specific the problem associated with old age like forgetfulness as an example, it also create the problem. The family also take it as normally , it is the old age illness, it happens when near to die like that…..This kind of attitude have also been seen in some member of the family, not for all but in some family which also be regarded as barrier.

And while talking about the facilitating factors, due to their sons or grandson who are studying, they themselves come to health facility to seek care and ask whether they have some mental illness as they have heard of it ….. As they have listen and get information from their children, they are self conscious and self aware which is good aspects.

Another one is, if the patients come them with our staff co-ordination we counsel them regarding to the mental health so that they help to identify the illness in their family or in society. This is also helping us.

Another is from the government….. Governments have also added the mental health related medicine in the essential drug list, exactly I don’t know about the all medicine. But the drugs have been added to the list.

Another thing is that, our Agrakhachi district in mental health ……….Arghakhachi, Gulmi and other 2/4 districts are the more severely affected by mental health related problem. Therefore, that the government gives priority to all but among them Arghakhachi and Gulmi are prioritized and the issues like requirement of more training to these area have been discussed . The medicine have also been added to the essential drug list which is also a facilitating factor.

This is how the facilitators and barriers are working

I: At institutional level, Did you found any facilitator or barrier for the patients ?

P: At institutional level, Government have tried to provide training or information related to mental health to the staff. But as being doctor during academic course, we have learned about the treatment and apply those. But all the staffs over here are also not as capable and are not trained and aware about the problem yet which may as barrier. Because if the awareness or training is provided about mental health to all the staff working in the Hospital or PHCCs then they can do additionally what they are doing right now. It he/she got training then they can be empowered and do better. Thus lack of training and awareness to staffs exists here. For us also, this much is not enough however we perform however other staff requires training.

While talking about other barrier ……. the problem is…. these all need to done by government with more efforts. Government has taken some efforts which are good things like providing medicine and awareness rising which is positive. It is good aspects. Whether the government does something or not, the staffs are being self up to date regarding new disease. Because now days, there are more prevalent of non communicable disease then communicable disease. Hence health staffs need to be self updated so that they could provide their knowledge to the patients. The staffs need to be self motivated to help their patients which is good aspects.

I: At community level, to get treated by the patients, what are the facilitating or barrier roles?

P: At community level, the facilitating factors staffs all are friendly, they have attitude to help their patients being self updated by going to community. While going to the community, there are many barriers or obstacles; it is more in rural area. Even giving the right medicine, they assume that the medicine is forcedly handed to them by us. This kind of attitude can be seen. As well as the mental health is neglected in community. Many cases are hidden to the household level, who does not reach to us. Additionally, when we go to the community, the trend of viewing negatively to us exists. Sometime when we counsel that the conditions is severe and require medication then they have belief that the such medicine should be consume for lifelong if started to take. These kinds of the belief play as barrier in the community. These are barrier and other things, as already told that lack of specific training and psycho therapist to counseling. Because of lack of the trained staff to counseling, counseling given by us is being insufficient. Another in the community level is, superstitious belief which as main barrier.

I: There are the barriers for the service delivery at the community level, what are the barriers for the utilization of the services?

P: For service utilization, although the specific mental health services are not available, we provide it as much as we can, but due to superstitious belief or other barriers they are not encourage to seek care. In spite of providing service to them, the community seems unwilling to take the services. If the member is from the educated family then they came themselves and ask about it otherwise they are not encouraged to take services. In contrast, they seek care from the Dhami / Jhakri rather than coming to us. This kind of mind-set is present in the community.

I: And at community level, have you found any supporting factors?

P: Supporting is also there…. How it is supporting is like if they are educated then they indicate us about some in the community might have the mental illness like by the FCHVs in the community reporting about the case seen in the community. We also orient them during training or monthly meeting that there might be mental illness in the community having some particular feature…. Then they also from their side make efforts to bring such neglected cases in the community to us. Likewise, other educated persons in the community also helped to provide information when meeting with us.

I: And for the community people, what are the supporting factor or barrier to utilize the mental health services at policy level?

P: At policy level, as I have told already that the more focus is given always to the higher centre regarding mental health services. Although it is not at the health post level, but at least at the PHCCs or District hospital level, it would be better if a trained counselor be available at those institutions. Because the medicine is already available which be provided by us but in the mental health if one gets good counseling in time then they wouldn't face problem in the future. Hence, if there is well trained counselor in the institution then it would be far better which can be improved from the policy level. Another, as focused policies are formed in different health conditions, mental health also require such focus while preparing policy. Also frequent visit to the community by counselor or doctor is requiring so that on the basis of what is happening in the community the policy could be developed which would be good. And while providing the services or medicines or drugs which are adding up also require further enhancement during policy development. The policy should be developing by focusing by all areas rather than single mental health. It needs to be prioritize like other areas.

I: Do you evaluated or diagnose the mental health conditions of the service users' visiting this institution?

P: At our level, we don't diagnose the patients as mental illness at the beginning, but according to the flow of the patients, we concern more towards the clinical like physical examination but if any cases arrive then we suspects regarding mental health then. As the patient does not share those at first visit hence it takes long time and ideally we also couldn't probe at such short time. Thus if some problem be identified then we call them for follow-up. This is the trend. But we don't perform the mental health examination in the OPD basis which needs to appreciate as our weakness. If some problem is identified, then I have planned to give them separate time as Arghakhachi is also victim of the mental health problem. Thus I am making the plan but unknown about its implementation as it is not possible due to many patients at the day time. It can be done differently if they have such illness.

I: In this health facility, are the currently available services to treat the mental illness adequate?

P: No. That is what I have already told that, not for mental health. We do what we know at our level; otherwise, government doesn't have support for us in mental health. It's been heard that support would be but we haven't receive, facilities are also limited.

I: And, what are the encouraging factors for delivering the mental health services for you??

P: How is that, the communicable diseases are prevailing since the earlier and nowadays, due to mental illness the people are living the life as disability. As I have been to Agrakhachi, I have seen and heard about disability and suicide. While hearing about such cases as the trend of going to the Dhami Jhakri and Mata is more prevalent in this district. After hearing such situation, I feel that these could be solved from our level; it could be prevented and removed. That is what I have seen. As if I could improve the situation then there would be satisfaction for myself as well. Thus these negative factors inspire me that I have to improve this factor. The main factor is that. Other are there would be the regular staff meeting, the issues are raised sometime but it is neglected as it is not regarded as a problem. There is good co-ordination so we can discuss that we can do something for it, what we can do to make the access of mental health like that…..

I: At your interpersonal level, what are the facilitators or the barriers for delivering the mental health services for you?

P: At personal level, I am helping them as per my knowledge and what I have learn about mental illness as my learning will help someone… And while talking about the barrier at individual level, in the community, while visiting the hospitals, they already have some attitudes or perception regarding what tests to do for them which need to be removed which have been as barrier. Other is that the patient flow also be high so I provide service as much from my side. I put efforts to remove such barrier from my level, and facilitators is my knowledge that it.

I: And at interpersonal level, are there any factors that facilitates or hiders in mental health service delivery please mention it

P: At interpersonal level, I have already told that, if someone is educated in family then they try to get service by building good interpersonal level and share their feelings. What happens in our community is that, the caretaker or person with mental illness require the discussion in separate place…. that privacy couldn't be maintained sometime. As there is already a kind of thinking or perception related to mental illness in community, there is need of privacy. While maintaining the privacy as well there is negative thoughts which remained as barrier.

I: At institutional level, have you found facilitators or barriers for providing the mental health services?

P: The facilitating factor at the institutional level is the co-ordination among staff so that we perform all the activities by discussing among us. The other is more barriers than being facilitator which might improve later. Like, the news of the medicine being available is just being heard which I mentioned you earlier. Only very limited medicines are available in institution which be remained as barrier. The lack of specific trained mental health related counselor or psycho therapist counselor staff also lead to problem as a doctor couldn't cure all the patients and if s/he can then also there might some inadequacy for counseling. So if someone does good counseling then it would be better. Lack of counselor is another barrier. And another barrier is …… how is in institutional level…… At community or rural area, access may be barrier as they might won't able to come at institution in spite of having mental illness. We couldn't bring them at institution as in urban area. Hence, due to being far away or due to distance, they are not able to seek care.

I: At community level, have you found facilitators or barriers for providing the mental health services?

P: Supporting factors are as…. The FCHVs in the community are supporting us. While having the regular meetings, they report us the cases they seen. They provide information what they know and I also provide them what I know. And they provide the information to the community. And as regard the barriers….. Barrier is that while providing such information to the community, they perceive it negatively. At the community there is old thinking and old people resides over there, because of them being less educated, while doing good for them as well, they thought that, We are forcing them to take medicine " *Yesle jabarjasti aausadhi vidauna khojeko*" like that. This kind of thinking has been as barrier.

I: And at policy level, to provide the mental health services, what you found as supporting or barrier factors?

P: For supporting….At policy level they are supporting by providing some medicine. In spite they couldn't provide all the medicine demanded but tried to provide the medicine available from their level. This also has been done so it can be positive. And another is…… While talking about negative aspects, I am here as physician, so If there is specific mental health counselor available for help then it would be better. And another is, all the OPD patients are kept at same place. They are recording and reporting is based on the same OPD register. Thus it would be better if there is specific different register could be maintained or separate recording and reporting system for the mental health which is neglected. So that any cases reach at the higher level and the cases be seen at the level if they reach to such level and the training and policy be developed. If they don't reach and no good reporting and recording then there would be difficult to develop policy which have been remained as barrier.

I: And at this facility to improve the mental health service delivery and service utilization what would you recommend?

P: The main things to improve at our PHC level is staff inadequacy. As I have already told you that, the availability of the trained specific counselor is needed. Either we available staff in the PHCC need to be trained. If the training for all staff couldn't be done at once than the turn wise training need to be provided. The materials, pamphlets, posters, etc for counseling need to be supplied as while only verbal counseling may not be enough, the counseling with the written notes and picture help to understand the message. Another is about medicine, as psychotropic medicines have their own limitation which need to be consider while prescribing, if the medicine be available sufficiently which can be provided from this level then it would be better. The next is, I am here as a Medical officer here, If the mental health specialist come at the rural level PHCCs sometime to provide the services then it would be more better. Another is that, the screening campaign for mental health can be organized by reaching to the community. While doing such screening program, it can be done by age wise so that many hidden cases can be identified by their symptoms and counseling can be done. We can bring out and report those hidden mental ill patients. These can be the aspects to improve. If this all could be done then I think it is enough.

I: Lastly, do you have something related to the research to tell which I have missed to ask and which you have to add up?

P: You are doing research in this … we are doing what we can do from our level. And you are also on the basis of your research and asking to many people, caretaker of the mental illness etc. What could be the result from your research, you do from your level. Only interviewing and asking question will have no use. You do whatever you can from your level. Improvement is possible. After all you are also responsible for making the policy in future. For that, from today you do what you can from your level that is my request for you. Lastly, whatever the weakness you see from my interview or other staffs’, please inform these at the higher level. That's all.

I: In spite of being busy schedule, you manage time for interview .Thank you so much

P: Ok thank you
