# Supplementary material for: Factors affecting utilization of mental health services from Primary Health Care (PHC) facilities of western hilly district of Nepal
Source: PLoS One. 2021 Apr 30;16(4):e0250694. doi: 10.1371/journal.pone.0250694 (PMC8087454; doi:10.1371/journal.pone.0250694)
Supplement: S4 Transcript — (DOCX) [file pone.0250694.s008.docx]

Interviewer: Please provide your brief introduction.

Participant: My name is D.G. I am AHW at …… Health Post.

I: What are the major health services provided by this health post?

P: The major health services provided by this health post are OPD services, safe motherhood services, CBIMNCI, immunization services; communities-based ORC clinics, health mother group meetings along with counseling and refer services for mentally ill patients who visit for health services.

I: What is the quality of health services provided from this health post?

P: We are trying to provide quality services as per our knowledge and the resources available. We do not have authority to purchase any resources and materials that are required for the health post. The rural municipality manages resources and materials, medicines, HMIS tools in timely manner and we as health service providers are trying our best to provide quality services.

I: What is the condition of mental health service provided by this health post?

P: People do not directly come with mental illness complaints but during the course of history taking we find some unrelated symptoms like sleeplessness and multiple symptoms and doubt it to be mental illness and counsel them. Recently I have heard that Province 5 had conducted training on mental health but there was no person from our health post that went for the training; maybe we get chance in near future. As there is development of new technology, if we identify different ways to provide mental health services effectively and if we are appropriately trained then in future, we can provide quality mental health services but till now we are limited to counseling and referring to mental health experts.

I: What are the factors that have supported mentally ill patients in the community to utilize mental health services from primary health care facilities?

P: (Interviewer coughs) There is not much awareness regarding mental health in the community but through mother’s group and FCHV some health issues are kept forward in each month’s meeting which may involve mental health issues as well like the family torture during pregnancy that may lead to mental illnesses. We ask mother’s group and FCHV to provide with proper counseling as well as create awareness in people about mental health. We orient FCHV every month in different topics and we provide case management techniques and ideas which they ask for regarding different cases present in their community. There has been no special program focused on mental health at community or in health facility, we identify mentally ill patients on the basis of their symptoms and counsel or refer them as per their condition.

I: What are the factors at individual level of mentally ill patients that affect mental health service utilization from this health post?

P: There is still presence of superstitious beliefs at villages regarding mental illnesses and people still visit traditional healers “Lama-Jhakri” thinking it may be due to witchcrafts “Boksi”, “Kholako tir laagyo”, or due to ill doings when performing some prayers to Gods. Here is a Lama present in the community where I work and we consult him as well and ask him to send those patients with mental illnesses to us so we could provide counsel and other services. When a person is not mentally satisfied that person cannot be cured properly so we ask the Lama to send such patients to us after his treatments. There is a famous Lama to whom people from different places come to visit, he performs different rituals like “Fukne Jharne” but he also asks mentally ill patients to visit health facilities for other treatments. The main factors hindering service utilization is lack of awareness in people and we have not been organizing any mass education program and proper individual and family counseling as well. There are still older peoples with traditional thinking who think mental illnesses as “boksi”, “kholako tiro” and visit traditional healers. If the disease gets worse then only do they bring the patient to the health facility for medical treatment. We at this level are very restricted in our roles and responsibilities so people at higher bodies should provide awareness program, mental health promotional activities so that mentally ill people can visit health facilities for their treatment. If we can generalize the mental issues by providing mass awareness in the community then it would be fruitful.

I: So you are trying to say that if there is awareness in people regarding mental health then they utilize mental health services?

P: Yes. If people have awareness regarding disease condition, state of disease, knowledge about the proper treatment centers then we can empower them to visit health facilities for treatment. If there is too much frustration regarding the disease as well as family pressure to visit Lama Jhakri for treatment then the condition might get severe. People in community do not have knowledge that it is also a disease and can get treated with medications. Many people in this community keep their mentally ill patients at home tying and not bringing them to health facilities for treatment. So due to lack of awareness mentally ill people are confined within house which may be severe and damage their life, I have seen such cases in one or two places that I have visited. Also, may be due to economic condition people do not visit health facilities for mental health services thinking it is in vain to treat such patients. So, in such cases economic support should be provided from the governing body identifying the socio-economic level of the patient. Main factors that I find affecting mental health service utilization is lack of consciousness and awareness regarding mental illnesses and the economic status, these factors restrict patient to utilize mental health care services.

I: Are there any factors at institutional level that have supported or hindered utilization of mental health services from this health post?

P: Husband of one of our service user after returning from abroad had mental illness, he used to remain isolated and not talk with anybody, so we counseled his wife to not worry and suggested her that medications would do her husband good and referred him to Butwal where he got treated and is well now. Though he still is less socialized but he speaks sometimes with us and his wife is happy in his improvements and wishes for involvement in business. So, we can only provide counseling services that has helped mentally ill people so people visit our health facility. As the husband came from abroad and was in communication with this health facility he could know about the disease and get treatment in time through our counseling, suggestions and referral and is now 70% free from mental illness and is still on regular medications.

I: At community level, are there any factors that support or hinder mental health service utilization from primary health care facilities?

P: To go to health facility?

I: Yes. Factors at community level that affect utilization of mental health services.

P: As the mental health services are not provided from our health post, it may act as hindrance for mental health service utilization. We have been providing counseling services, suggestions and referral services only but it would have been better if we had training on mental health or if it was possible to call an expert for 2 to 3 days per week to treat mentally ill patients, it would do a lot better to mentally ill people. Sometimes due to economic reasons, sometimes due to lack of awareness, sometimes due to lack of consciousness and sometimes due to traditional concepts and thinking service utilization is affected a lot. Due to traditional thinking, people neglect the disease or go for traditional healers to perform various rituals like “boka chadaune”, “Jharfuk” which we have not been able to minimize and thus are creating hindrance to mental health service utilization. The main factor is lack of awareness which has hindered service utilization. Factors affecting should be searched for at different municipalities. We do not have such tools and programs for mental health awareness but also, we are providing counseling services and suggesting referrals to higher level facilities. If the higher authority provides mental health care tools and awareness program the issue will be solved.

I: So, there are no any factors at policy level that has supported or hindered mental health service utilization?

P: No, there has not been any. From the time I started providing health care services, we have to provide services as per our knowledge from our study, there are no any refreshers. Also there has been no monitoring from higher levels, only providing training and making policy is not appropriate if there is no monitoring or supervision. If there is good monitoring and supervision of implementation of services and programs after providing trainings and resources then only can there be proper mental illness treatment.

I: Do you assess or screen patients for mental illnesses in this health post?

P: As the patients come to OPD we take detail history of the patient that includes family history, personal history, education, occupation, family disease, family status etc as well as observe them carefully and if they complain of symptoms of mental illness or ask for medicines to help them sleep then we provide medications if available with us for prevention but if the illness is severe than we counsel them and refer them to higher centers. There are some sedatives medicines that we are allowed to prescribe and if there is demand for those medicines, we provide the patients with those medicines otherwise we just counsel and refer. We do not have any specific mental health service except counseling and referral.

I: What are the factors at your individual level that support or hinder you to deliver mental health services?

P: There is not any hindrance at individual level. As I have told you earlier, patients first visit to Lama and he asks them to visit health facilities as well and they come here. People do not come to health facility without visiting the Lama and accepting tika of Kharani there (laughs). After they arrive at health facility, we provide proper suggestions and counseling and advise them to go to higher centers as per requirement. We also say them that they can visit traditional healers but they should not miss medicines and we sometimes suggest higher centers for other ailments which aid referral for mental illnesses as well.

I: I wished to know whether there are any factors that supported or hindered you for service delivery at your own individual level.

P: You mean to say to the patient?

I: No. Factors that made it easier or difficult for you to deliver mental health services?

P: There is no such factor that support or hinder me to deliver mental health services. Family and community bring mentally ill patients to health facility that is support provided by them. We also counsel neighbors in case of some difficulties for the family members to bring the patient. If someone finds it difficult, we also suggest them to go to higher centers for medications. So, there are not any specific difficulties or hindrances.

I: Are there any factors at institutional level that has supported or hindered you to provide mental health services?

P: There is no factor that hinders at institutional level. All the staffs in the health post discuss about the case and take decision for the patient after looking every fact. There is established coordination among us like if there is something that I do not know about then the senior staffs help in such cases, also the management committee supports us when required. Though people at higher authority say about supports but it is not seen, may be because of lack of balance or due to lack of resources or due to lack of budget distribution from policy level. There is no factor hindering the service delivery and we as well have been providing services as per our ability, skill and knowledge with the available resources. We discuss cases if it is suspicious and work as a team in the health post for the proper management or referral, whatever is required to the patient. There are no any hindrances. Everyone only say that they will provide support but we as a team have been providing counseling in this limited resource environment in case of mentally ill patients.

I: Are there any factors at community level that has supported or hindered you to provide mental health services?

P: There are no factors at community level. If there are any cases of mental illnesses in community and if they are not visiting health facility for treatment, we ask representatives to suggest such people to visit health facility so that we can treat them in the health post if possible or refer them. There was a patient who used to get irritated always and after counseling he improved without need for management from specialized doctors. There are many hidden cases in community or cases who do not wish to visit health facilities, so for such cases we ask representatives to counsel them to visit health facilities because if the patients do not get proper counseling and treatment in time severity of the disease increases and their life will be damaged. FCHV also discuss about mentally ill patients in the community during monthly reporting if they suspect anyone having such diseases. Representatives from mothers’ group have also provided support. Women’s network is also present in the community which acts on household violence and report them to our health post. Community leaders like teachers also help by providing suggestions for treatment in health facility. So, these all have been providing support in the community and have positive concept regarding mental illness and its treatment. But, in families with traditional concept, it is hard to make them understand and change their belief regarding mental illnesses. Younger generation understands, but older generation is resistant to the treatment of mental illness at health facilities and prefers traditional healers and has been acting as barrier.

I: Are there any factors at policy level that has supported or hindered you to provide mental health services?

P: There is no hindrance from policy level. But higher authority has been providing oral statements for support during meetings but have not implemented in actions. Recently some 26/27 health staffs went for training in mental health from our district which may be the start of implementation of programs and activities for mental health. The start from training must now lead to establishment of Mental Health Care center at our rural municipality as a referral center and providing mental health care from specialist doctors and treating mentally ill patients so that people need not travel far for treatment. Such initiatives should have been from policy level but there is not, also there are not any hindrances. The training that has been given may bring new tools and norms, research may bring some changes as the health sector is never stationary and we need to be regularly updated so there must be ample resources and a mental health center which I positively think of. There is no hindrance, no such directives as not treating patients. I think that Province 5 is the only province that is providing training and as health staffs get trainings, they will be expert in mental health and can provide services within this rural municipality or else can refer to best place for treatment which may be counted as policy level support. There is positive hint as those who have been to the training may share with us their experience and skill and we may also be able to manage cases of mental illness within our health post.

I: What suggestions do you like to give in order to improve service utilization and delivery from this health post or primary health care facilities?

P: From institution to the patients?

I: What changes or improvements are required in order for mental health service utilization and delivery…?

P: (Interrupting) At first health service provider should be well trained and expert on the subject, then the resources required should be present. Personal counseling and family counseling though are available at health facility but awareness should be created at community level, people should be visited in their home and counseled to get mental illnesses’ treatment at health facilities. Also, there should be availability of separate room for counseling as well as male health staff for treating male and female health staff for treating female mentally ill patients should be present. If it happens then we won’t require sending mentally ill patients to any other places for treatment. It would be more effective if there is provision of mental health services from all health facilities as well as a mental health care center from where specialized services could be provided from specialist either in person or from phone “Hello Sewa” which would minimize problems of many people from remote places. But for these all at first there should be expertise and skill in health care service providers and willingness to practice those skills. Higher authority should also provide regular monitoring and supervision to find out whether there is proper implementation of services or not.

I: At last if there is anything that I missed to ask or you wish to add related to this study/research, you may please add.

P: We all, from bureaucracy level to grass root level in health, must work hard in the case of mental health similar as working for full immunization. We succeeded in creating awareness in people regarding immunization and there have been many municipalities that are declared full immunization or near to full immunization, similar awareness should be created regarding mental health and its treatment. We should all work to make institutional treatment of mental illnesses 100%, which is a possible task. As there can arise serious problem in lack of vaccines for immunization, similarly if services are not provided for mental illnesses properly then may cause different problems, thus there should be regular monitoring and supervision within catchment area and health facility should be punished in case of any negligence. Only providing policy and plan without implementation will do no good for mental health of the community.

I: Thank you for providing your time for this interview despite of your busy schedule.

P: I would like to thank you as well for providing me the opportunity to talk about mental health of this rural municipality.

I: Thank you.
