# Supplementary material for: Factors affecting utilization of mental health services from Primary Health Care (PHC) facilities of western hilly district of Nepal
Source: PLoS One. 2021 Apr 30;16(4):e0250694. doi: 10.1371/journal.pone.0250694 (PMC8087454; doi:10.1371/journal.pone.0250694)
Supplement: S5 Transcript — (DOCX) [file pone.0250694.s009.docx]

Interviewer: Please, give me your Introduction?

Participant: I'm SL B.K

I: SL B.K .What is your relation with the patient?

P: Wife.

I: Patient is your wife?

P: Yes.

I: Did you know anything about mental problem before your wife had mental illness?

P: Umm… No Sir, not initially.

I: After?

P: After umm symptoms were seen. She used to speak loud and gave speeches like a political leader. She had no senses and used to throw away things unnecessarily.

I: It means that you didn’t know anything about mental problem initially?

P: She had been to her mother's house for taking part in a function. After returning from mother house she started speaking unnecessary things, she had no senses then and had abnormal movements in body. We took her to traditional healer for “Jharfuk” and she became fine that day. Again after 12 or 13 years this happened with her again and we took her for “Jharfuk” but it did not make her well. Everyone suggested that I took her Bhairahawa for treatment, so I went there and doctor gave some medicine to her which made her little better. The doctor asked to visit him regularly for check-ups and medicines. The doctor who sits at the OPD at Bhairahawa comes to Sandhikharka every second Saturday of month and we go there for checkup and doctor give medicines and she takes those medicines on daily basis. She has become well because of those medicines.

I: And, in our society how do people perceive mental illnesses?

P: Now? About patient Sir?

I: No, no. Perception of people in the society regarding mental problems.

P: Umm… They say the she is all good.

I: How people perceive mental problems in our society? I mean if they take it as good or as bad?

P: Mental illness is not good sir (Laughs).

I: I wanted to know how people perceive mental illnesses in the society.

P: They say “dimag bigreko”, “hos naaaune”, “sor naaune” for people suffering from mental problems.

I: So, the people in society take mental illnesses as any other illnesses?

P: (little bit confused) Educated people…. Different people talk differently about mental illnesses.

I: Yes, I am asking the same question. Please tell me what different people think about mental illnesses?

P: What people say is, it causes loss of senses “sor naaune”, is a mental problem, “dimag bogreko”.

I: Umm… What do you think causes mental illnesses?

P: Causes for mental illness… I think… She takes a lot of tension (stress).

I: And?

P: She also used to drink alcohol Sir which used to cause severity of symptoms. Doctor asked her not to drink alcohol but she still drinks alcohol every 10^th^ or 15^th^ day. She only drinks alcohol with friends or if someone asks her; she does not drink alcohol alone like she did earlier.

I: Any other causes of mental illness?

P: It is not due to anything else Sir.

I: Please share your experiences and feelings after start of symptoms of mental illness in your wife till today?

P: About mental problem?

I: As symptoms of mental illness were seen…

P: (Interrupting) About what she said at that time?

I: No. Initially some symptoms of mental illness might have been seen. So, I wished to know, from the start of the symptoms till today what are your experiences, which places do you visit, any difficulties, any helps, everything.

P: It was very difficult Sir. She used to refuse to go to hospital saying she would be fine by self. So, one day I called an ambulance and asked her to come with me to daughter’s house and from there took her to doctor for treatment.

I: Which places did you visit to identify the illness?

P: I had already known that it was mental illness Sir. Her talks used to be irrational, she used to talk without senses, so I knew it was mental illness and took to the doctor immediately.

I: So you directly took her to doctor for treatment?

P: Yes, directly to the doctor. At first I took her to traditional healer for “Jharfuk” which did not cure her, so I took her to doctor for mental illnesses.

I: Okay. You took her to traditional healer for “Jharfuk” first then to the doctor. So, what were the factors that supported you or hindered you for treatment of your wife?

P: Support… I used all my money and searched for money from different places.

I: What hindrances did you face?

P: The main hindrance was that I had to take care of her all the time. I used to look after her whereabouts and her actions. She used to have no senses, not eat food, talk unnecessarily and used to keep on thinking what other said to her all the time and take tension. Thus I understood it is mental problem and took her to Bhairahawa for check-up at OPD where the doctor checked her and prescribed some medicines. She has improved by taking that medicines Sir.

I: What factors affected you to utilize mental health services for your wife?

P: Those were the factors Sir, what more…(Laughs).

I: There may be many factors that affect service utilization. Were there any factors that supported or hindered you for mental health service utilization?

P: About support…umm… Everyone told me to take my wife to hospital so I took her to hospital for her treatment.

I: (Interrupting) People in society…

P: Yes. People in society said that she won’t get better by taking her to traditional healers so I took her to hospital. (Laughs)

I: Were there any factors at individual level that affected mental health service utilization?

P: No one provided me hindrances.

I: Were there any factors that supported or hindered mental health service utilization at your individual level?

P: Mostly she used to reject to go to hospital for treatment. Sometimes she used to get ready to go and then suddenly used to change her decision. That was what caused me some difficulties.

I: So, there was nothing else that made it difficult to go for treatment?

P: Yes. She used to be indecisive before but as she took medicines for 1 month she improved and used to ask to go for check-ups. She is fine now. She has grown fat now, which may be due to the medicines and also she sleeps more. She wakes up at 8 or 8:30 in the morning after taking medicines at night.

I: You took your wife to traditional healers for “Jharfuk” and “herauna” and also to Bhairahawa for treatment. Did not you take your wife to public health facilities within this municipality for treatment of her mental illness?

P: I did not take her to public health facilities Sir.

I: So, you did not take her to public health facilities for treatment?

P: I had done an insurance Sir, so they sent us to Dhorbas, Palpa. At Dhorbas there will be different doctor and the doctor will examine in their own way, and as the medicines she was taking were doing her good, I feared that the disease may return if the medicines are changed at Dhorbas, so I did not take her there.

I: So you did not take her there?

P: Yes Sir. The doctor who had initially assessed her is checking her regularly and the medicines that he provides is improving my wife’s condition.

I: What were the reasons for you to not take your wife to public health facilities initially?

P: If the disease had been cough and cold or some other diseases then I would have taken her to public health facilities Sir, but as her illness was mental illness it was no good taking her there.

I: Why?

P: There are no doctors for mental illnesses here at public health facilities so I did not take my wife there for treatment.

I: And?

P: That’s all Sir (Laughs).

I: So you did not visit public health facilities because there were no doctors for mental illnesses?

P: Yes.

I: As you had done insurance, health care workers at public health facility had referred you to other higher facilities, why did not you go there for treatment then?

P: Yes they had referred Sir, but I did not go as the doctor would change and the way of medications and medicine as well. As the medicines she has been taking was improving her condition, so I did not go to other place for treatment fearing that change in medicine may deteriorate her.

I: So, you did not take your wife to public health facilities within this municipality as there were no doctors for treating mental illnesses here?

P: Yes Sir, I did not take her to public health facilities because there were no doctors.

I: Any factors else than those?

P: No Sir.

I: Nothing else?

P: I had already identified the disease and it would have done no good had I taken her to public health facilities as there were no doctors for mental illnesses at public health facilities. It would have done no help (Laughs). So, in spite of spending money unnecessarily, I directly went to hospital where her illness could be treated.

I: At community level, were there any factors that supported or hindered you utilizing mental health services for treatment of your wife?

P: There were no any hindrance from anyone in the community and about support… they would provide loan in case of need which I used to return after some time.

I: At policy level, were there any factors that supported or hindered mental health service utilization for treatment of your wife?

P: No Sir, nothing from policy level.

I: No factors affecting at policy level for treatment?

P: I had an insurance Sir, the one that costs Rs 2500, they referred to Dhorbas for further treatment. If they had referred me to the same doctor who checked my wife at first then it would have helped me a lot. I did not take her to Palpa as there will be different doctor with different treatment style and will prescribe different medicines than which she is taking now. So I did not took her there.

I: Please correct me if I am mistake somewhere about the things that you have told me till now. At first you went to traditional healers then you yourself identified your wife having mental illness and took her to Bhairahawa for treatment and not at public health facilities as there were no doctors for treatment of mental illnesses.

P: I took her to a traditional healer (Buddha Lama) at Haraiya for “Jharfuk”. He told me to keep my wife there for 7 days in order to cure her, and also said that doctors would take 15 days to cure her. I did not believe his methods Sir, he would keep their and say to perform many rituals but at doctors they would provide medicines and show the differences. So, I took my wife to Bhairahawa for treatment.

I: Did the traditional healer ask you to go to doctors for 15 days or did he say 7 days were required for him to cure and 15 days for a doctor?

P: Traditional healer said that he required 7 days for treatment for which the doctor would take 15 days. I did not have belief in his words, trusted the doctors and went to doctors for treatment. After taking medicines prescribed by the doctor she has become well.

I: So you visit the doctor at Sandhikharka now?

P: Yes, he comes to Sandhikharka at second Saturdays of every month. He provides medicines for 1 or 2 months. Initially he had increased the medicines but now he has reduced the medicines.

I: So, you go to clinic for mental health services and not to public health facilities, isn’t so?

P: Yes, because the doctor comes to the clinic to provide mental health services.

I: So, were there any factors that supported or hindered mental health service utilization from the clinic?

P: At clinic first we have to note down our name on register, must pay Rs. 450 as doctor’s fee and should buy medicines by self, that’s all.

I: What improvements do you suggest in order for proper utilization and delivery of mental health services from nearby public health facilities like Health Post, PHCC?

P: Who me…(Confused)

I: What improvements do you think should be made so that there could be provision of mental health services from public health facilities and people of community would go to those facilities for treatment of their mental illness?

P: At Health Post they note down the name, provide some medicines and sent or refer to some other places if those medicines do not improve the illness (Laughs) and we have to go to referred places then.

I: What suggestions would you give to improve in order to provide mental health services from public health facilities?

P: Okay, about improvements… They provide medicines and if there is no improvement then they send to other higher centers.

I: Okay, they sent to other places if no improvement is seen. I wished to know about your suggestions for improvements such that mental health services could be provided by public health facilities like Health Posts.

P: There is nothing at public health facilities.

I: Okay. Since there is nothing there, what improvements should be done?

P: (Confused) We should do… We go there, they write our name and doctor checks us. Then the doctor provides medicines and if the medicines do not help improve then they suggest to go to Butwal or Bhairahawa foe further treatment.

I: Okay they ask you to go to Butwal and Bhairahawa in case of no improvements; I wanted to know what your suggestions are so that mental health services could be provided from public health facilities within this municipality. What should be done for the treatment to be possible at public health facilities?

P: At public health facilities they ask to buy medicines from outside the health facilities in case of serious illnesses. I think that those medicines should be provided from within the health facility.

I: And?

P: That’s it Sir. (Laughs)

I: Anything else?

P: No one is equal here Sir, some are rich and some are very poor who cannot even earn that can provide them food to eat once a day. Such poor patients cannot buy medicines from outside, so I suggest that the medicines should be available within the health facility for such poor patients.

I: Any other suggestions?

P: That’s it Sir. We have to buy medicines from outside which should have been available within public health facility. So I suggest that those medicines should be available from within health facilities.

I: If there…?

P: (Interrupting) They write medicines and ask to buy medicines from the medicals outside so we have to buy those medicines from medicals and take those medicines.

I: So, you are saying that if medicines for mental illnesses were available within public health facilities then people would go to those facilities for treatment of mental illnesses?

P: I have not seen people with mental illnesses go to public health facilities for treatment of their illness.

I: Do you think that people will go to public health facilities for treatment if medicines for mental illnesses are available there?

P: Yes Sir.

I: And they won’t go to clinics?

P: No Sir. They won’t go to clinics and will go to public health facilities then. If the medicines are available at public health facilities then they would not visit other places Sir, regularly take medicines from public health facilities.

I: People won’t even go to clinics for identification of disease?

P: If the services are available at public health facilities why would people go to different places Sir.

(Silence for some seconds)

I: If there is something that I missed to ask regarding the research...

P: (Interrupting) Sir, if anyone is enrolled in the insurance and is referred to other centers for treatment, only the costs for medicine is covered and not of transportation for which we have to pay self, so it is not of help. If the cost adds up to Rs 3000 here then it would be Rs 7000 there, it would be difficult to pay those 7000 Sir. They had referred me to Dhorbas which would be much costlier to me. If there were medicines available at Health Posts then I would not have visited other doctors at clinic.

(Silence for some seconds)

I: If there is something that I missed to ask or you wish to add regarding the research, please you may add.

P: It’s enough Sir. That’s it Sir. If there are small health problems then they provide medicines from within the Health Post, but if there are some bigger health problems then they prescribe medicines from medicals saying there are no medicines in the Health Post for that disease. People having money can buy those medicines but those who are poor cannot and have to die in vain. These issues must also be considered by Health Post or public health facilities. That’s all Sir. (Laughs)

I: Thanks a lot for providing time for this interview.

P: (Laughs) Namaskar.

I: Namaskar.
