# Supplementary material for: Factors affecting utilization of mental health services from Primary Health Care (PHC) facilities of western hilly district of Nepal
Source: PLoS One. 2021 Apr 30;16(4):e0250694. doi: 10.1371/journal.pone.0250694 (PMC8087454; doi:10.1371/journal.pone.0250694)
Supplement: S6 Transcript — (DOCX) [file pone.0250694.s010.docx]

Interviewer: Please introduce yourself.

Participant: Namaskar, my name is M.N., Hansapur, Malarani-4 Bamruk. Patient is my daughter.

I: So the patient is your daughter?

P: Yes.

I: Did you know anything about mental illnesses before you daughter was mentally ill?

P: Yes, my mother in law also had same disease.

I: What things did you know about mental illnesses?

P: Mother in law also had same pattern of disease, fall down suddenly while cooking, while talking to other. Later she was medicated and now she is fine. I think same disease transmitted to my daughter.

I: You think that it may have transmitted to your daughter?

P: Yes, I think that it may have transmitted via food or anything else what my mother in law used, but the doctor said it was not.

I: How do people in society perceive people with such diseases?

P: They think that these illnesses are communicable and they call such people as ”pagal”.

I: In one word, what is the perception of society towards mental disease, good or bad?

P: Bad one. They have bad perception.

I: Why bad perception?

P: They think these diseases are communicable one so.

I: They think it as communicable disease so?

P: Yes, they say it is communicable, it is very dangerous, do not touch the patient, do not walk with them, do not have the meal on same plate or you may get the disease.

I: What do you think causes these diseases?

P: I think that it may be due to lack of nutrition, unbalanced diet and lack of other things.

I: Lack of food and nutrition?

P: Yes.

I: What are your experiences from the start of symptoms till date during mental health service utilization?

P: At first, we thought it may be due to physical weakness so we gave balanced diet, nothing improved. Then we took her to Aamda hospital at Butwal where doctor said it is seizure, it’s because of malnutrition and lack of calcium so they gave calcium supplements. Even after 15 days of treatment, she was not improved. Again she was provided with treatment for 10 days. But the symptoms came almost daily or every 5-7 days, the disease has not been cured.

I: What places did you visit for diagnosis of the disease?

P: Twice to Aamda hospital and now in this clinic.

I: Did not you go to any other places for treatment of the disease?

P: Yes, we went to traditional healers for “herauna”.

I: Could you please say something about that?

P: At the traditional healers they said “laageko ho” and “laas” or something.

I: Why did you visit traditional healer? Did you visit to traditional healer or hospital at first?

P: First we visited hospital, during the course of treatment one of my relative gave advice to go to traditional healers for “herauna” so we visit them.

I: Would you please elaborate?

P: At traditional healers’ (Dhamis’) place, some told it may be due to “raas uthayera rakheko” and some told it as “laageko” but the symptoms used to return.

I: You visited traditional healer only once?

P: We visit one traditional healer continuously for more than one month and another traditional healer only once.

I: At first you visited Butwal then traditional healers and again to Butwal?

P: Yes. After 15 days.

I: Is your daughter using medicines given from Butwal now?

P: Yes. My daughter’s symptoms were being continuous recently so I gave her higher dose of medicine and the symptoms subsided. As my daughter hasn’t been cured after medications for 3-4 months, we visited here for further check-ups.

I: So you increased the dose of medicine by yourself?

P: Yes. As per the doctor… the doctor had suggested increasing the dose a bit if there were no improvements and bringing her back for further examination if the condition gets worse. My daughter does not take the medicine properly and sometimes the medicine spills so I increased the dose. Sometimes the symptoms do not appear for 10-15 days and again starts.

I: Were there any factors that supported or hindered you to utilize mental health services at different places?

P: There was nothing.

I: So there were no factors that supported treatment of your daughter?

P: I could not understand what you asked.

I: Any kind of support that you received during treatment of your daughter?

P: No, we did not ask for any support from anyone. My uncle was with me when we went to Aamda Hospital as people suggested that it provided good treatment.

I: Did you face any obstacles during treatment of your daughter?

P: When we first visited Aamda, the hospital was closed as it was Sunday so stayed there that day and the check up was done next morning.

I: Any other factors that hindered service utilization?

P: No other factors hindered service utilization.

I: Did you visit any public health facilities for treatment of your daughter’s illness?

P: No.

I: But why?

P: “Yetikai” (Simply).

I: Simply, is it? Why so?

P: My mother in law was cured after receiving treatment here. And people also said that there was no treatment of mental diseases in government hospital.

I: Who said so?

P: People of village, our neighbors said there were no psychiatric doctors in government hospital.

I: So people in village said there was no service for mental illnesses in public health facilities?

P: Yes. They said that there were no doctors for treatment of mental illnesses. As the symptoms were similar to that of my mother-in-law so we knew that it was mental illness and thus did not take my daughter to public health facilities of our rural municipality.

I: Were there any factors at community level that supported or hindered the treatment?

P: A person in the village asked me to visit Aamda Hospital; he said there were doctors and facility for this disease and asked us to take some contact numbers. But as our uncle was there who knew about the hospital and had previous experiences so we did not require that help.

I: Any hindrances?

P: No

I: Any other factors that supported or hindered service utilization that you remember?

P: Nothing.

I: So the only hindrance was that the hospital was closed on Sunday and the uncle who helped was supportive; any other factors that supported or hindered service utilization?

P: No, there was nothing else. An uncle from our village had hotel near the hospital, we stayed there and he provided with suggestions regarding treatments.

I: And also you did not visit public health facilities because there were no treatments of mental illnesses?

P: Yes, not at our village and neither at Sandhikharka. We maintained nutritious food at home and directly took her to Butwal for treatment.

I: At first you thought it might be due to food so maintained it at home, then you took her to Butwal when she did not improved, after that you took her to traditional healers then again to Butwal?

P: Yes.

I: What are your suggestions for improvements in the public health facilities so that there is mental health service utilization from those facilities?

P: There should be availability of doctors and medicines. Doctors for treatment of mental illnesses should at least be available on monthly basis if not on daily basis.

I: There should be availablility of doctors?

P: Yes. It would be a lot easier.

I: And the medicines as well?

P: Yes, the medicines for mental illnesses should also be available.

I: Any other things that you want to share that I may have missed to ask or that you wish to add regarding mental illness that may be helpful for this research?

P: Is this disease communicable or non-communicable?

I: You are asking me to response?

P: Yes.

I: We would talk about that after the interview. For now, if there is something that you would like to add regarding mental illness that may aid my research, please you can add.

P: There is nothing more to add.

I: Okay. Thanks a lot for providing your time for this interview.

P: Okay.
