# Supplementary material for: Factors affecting utilization of mental health services from Primary Health Care (PHC) facilities of western hilly district of Nepal
Source: PLoS One. 2021 Apr 30;16(4):e0250694. doi: 10.1371/journal.pone.0250694 (PMC8087454; doi:10.1371/journal.pone.0250694)
Supplement: S7 Transcript — (DOCX) [file pone.0250694.s011.docx]

Interviewer: Please provide your introduction.

Participant: What should I say? My name is U.D.G.

I: U.D.G. So you are patient?

P: Yes, I am.

I: So you have mental illness?

P: I do not know about mental illness but I have severe problems in my stomach.

I: Have you been taking medicines for the disease?

P: Yes I am.

I: Did you know anything about mental illnesses before you had mental illness?

P: No, I did not.

I: So, you did not know anything?

P: No.

I: Nothing at all?

P: No I did not know how it happens and what to do about it.

I: And now do you know what causes mental illness?

P: I do not know how the disease started.

I: So you don’t know?

P: At first I started dislike for food, I could not sleep, I was restless and I had stomach pain a lot. People say that even gastric makes you go crazy, so I am regularly taking medicines for gastric which has provided some relief. (Pauses) It may be caused due to improper food habit for I, I do not what may be the cause for others. At first there is dislikes for food, then sleeplessness, followed by stomach pain with burning sensation throughout the body with “jhamjhamaune” and “silka hanne”.

I: How do people in society perceive mental illnesses?

P: People say that if we do not think a lot and do not worry much then it reduces chances of getting mental illnesses.

I: So, people say not thinking a lot and not worrying reduces chances of mental illnesses?

P: Yes.

I: How do people in society perceive patients of mental illnesses?

P: “Hepchhan hela garchhan tyei ta ho kyare”. They talk bad about people with mental illnesses. They also say she has gone crazy “boula” and is a witch “boksini”. The cause of my mental illness is different worries that I have but people talk about witchcrafts as reason for my illness and ask people not to talk to me. People in society perceive patients of mental illness very badly. Some people may be understanding and show sympathy but many talk badly about people with mental illnesses.

I: What type of people in society speaks well and what type of people speak badly about people suffering with mental illnesses?

P: I do not know what type of people.

I: You told that some people show sympathy while others speak badly, what type of people are they…?

P: (Interrupting) Some may have empathy and speak well and the rest have their own thinking regarding the disease, they see things in their own way.

I: Can you share your experiences from the start of the symptoms till now for treatment of this disease? (Silence for some time) What places did you visit for treatment of this disease?

P: At first we went to Bhairahawa during 2060 BS and the symptoms were not present then after. The symptoms of “jhumjhum” started from Asadh of previous year which is still present, I am not completely cured. Sometimes I feel comfortable and fine but sometimes the symptoms are more severe. So, due to such symptoms and fear of severe illness/death I ask my daughter-in-law and my neighbors to take me for treatment. There is increase of fear in me after 2060 BS.

I: So you directly went to private hospital for treatment?

P: Yes, initially I was taken to Bhairahawa in 2060 BS. I knew nothing and was completely senseless then. They had treated me with current (ECT) there and I was well then after. I had not taken any medicines and was doing well at my household chores, managing livestock properly as well as doing some business. But from Asadh of previous year, “jhumjhum” began with severe pain in stomach, dislikes for food and feeling of current “siringa” starting from foot moving to head. Sometimes I feel fine and do my works, sometimes I feel unwell and sleep. It has been such since last Asadh.

I: So you did not go to any places else and directly go to private hospital at Bhairahawa?

P: No, directly went to Bhairahawa.

I: You did not go to traditional healers for “dekhai herai” as well?

P: No. directly went to Bhairahawa and was admitted there for 22 days. I returned completely well after 22 days of treatment there.

I: Are there any factors that support or hinder mental health service utilization?

P: Support is that people in community bring me here for check-up. There has been no any other support or hindrance as such.

I: Any hindrances that you faced while going for treatment?

P: No there were not.

I: So, there were no any barriers or hindrances?

P: What hindrances am I to say?(Another woman replying to the question and participant following her lead) Yes, it was difficult for my daughter to find good place and required money for my treatment. She is also somewhat sad always. I only have her with me. (Another woman adding to participant’s response) I did not feel any difficulty as I was senseless; my daughter had to face many problems then. I searched this clinic by myself but previously I need not had to do such things.

I: What factors hinder you now?

P: (Laughs) It was difficult to find clinic providing treatment of mental illness. As I had no money in hand so I had to ask loan from others which I said would return as I be well and either by earning or buy selling properties.

I: So there is problem of money?

P: Yes.

I: So, lack of money acts as a barrier?

P: Yes, it has. It is so difficult, if left untreated severity of the disease increases, if treated there is issue of money. Treatment is too much costly here, even the medicines.

I: Did you not visit the nearby public health facilities like Health Post for treatment of mental illness?

P: No, I have not.

I: There are Health Posts in your municipality, aren’t there?

P: Yes, there are.

I: Why did not you go there for treatment of your illness?

P: People said that there is no service for mental illnesses at public health facilities so I did not go there.

I: Who said so?

P: There are some relatives working in health post who said that they do not provide medicines for mental illnesses from health post so I never went there for treatment.

I: So, as medicines for mental illnesses are not present…?

P: (Interrupting) Yes, as medicines were not available at Health Post so I did not go. There are many doctors among our relatives and in the village, so we ask them for medicines and they say they can provide medicines for gastric, body ache, jhamjham etc. and provide them except for one medicine.

I: So your relatives work at Health Post?

P: (Another woman responding and participant following her) No, they have private clinics and I ask them for medicines when I am unwell… (The lady again responds and participant follows) A relative is nearby us so I ask him for treatments and suggestions… I am sweating (laughs). I ask him whether he can provide me medicines that I was prescribed from check-up at different place and he says he can except one medicine.

I: So the medicine is not available at health post as well?

P: Yes.

I: Is it because the medicine is not present at Health Post or because he cannot give the medicine?

P: He says that he cannot give the medicine.

I: So he says he cannot give those medicines?

P: Yes. He says he cannot give that one medicine. I ask him to provide me saying I feel very unwell but he does not provide that medicine.

Another woman questioning the participant: Did you go to our Health Post? Did you ask medicine from Health Post?

P: Hmm… No. Not at Health Post. (Pauses) They do not provide medicine at Health Post by just asking for it and I have not been there for medicine as well. When going at local clinic he provides all other medicines except one which is to control symptoms of mental illness. I do not know why he does not give the medicine, I guess it may be because it may cause difficulty to the patient, but he does not provide that medicine.

I: At policy level, are there any factors that supported or hindered mental health service utilization?

P: No, I have not.

I: There is nothing, neither support nor hindrance?

P: No.

I: At your individual level are there any factors that supported or hindered treatment of mental illness?

P: Individual… I do not have any source of income or anyone to provide me with money. I have a buffalo and I sell ghee and milk, that’s all I have. (Another woman speaks and participant follows) I have no one and nothing to support me.

I: And from community level are there any factors that support or hinder?

P: No there are not any.

I: So, there is neither support nor hindrance?

P: Yes.

I: What suggestions do you give in order for improvements in nearby Health Post for mental health service utilization?

P: Umm what to say… (Another woman responds and participant follows) Yes, it would have been a lot easier if the services were available at Health Post.

I: What improvements should be done? (Pause) As you have mentioned that they do not provide medicines, which is not good for treatment and patient, so what type of improvements should be done?

P: (Another woman providing answer throughout the question to the participant to say) If the medicines were provided at Health Post then we need not come here. They do not provide medicines at Health Post so we come here. They provide 2 to 4 medicines for simple diseases but not of mental illness. (Another woman still speaking that doctors and medicines for mental illnesses if present at Healh Post would do them a lot better).

I: Any other suggestions?

P: What more shall I add.

I: So, you say that if there is availability of medicines and doctors for mental illnesses at Health Post then you would go to Health Post for treatment of your mental illness?

P: Yes I would go if it was so. But they only provide Diegene tablets and send which cannot cure mental illness.

I: At last if there were anything that I missed to ask or something that you would like to add regarding this research, please you can add.

P: What should I say? Nothing comes up in mind now (Laughs), later it may. We need facilities but we cannot get that, we are always spending.

I: What type of facilities that you are talking about?

P: Of everything.

I: What everything?

P: (Thinks for some time) What to say, I do not know anything.

I: You said that if there were facilities of everything then it would be a lot easier for you. What type of facilities did you wished for?

P: I wish to live well with proper food and clothes. I do not have sons so I now wish that I live healthy.

I: Thanks a lot for providing time for this interview.
