# Supplementary material for: Factors affecting utilization of mental health services from Primary Health Care (PHC) facilities of western hilly district of Nepal
Source: PLoS One. 2021 Apr 30;16(4):e0250694. doi: 10.1371/journal.pone.0250694 (PMC8087454; doi:10.1371/journal.pone.0250694)
Supplement: Attachment — Submitted filename: Response to Reviewers.docx [file pone.0250694.s012.docx]

We would like to thank the reviewer for reviewing our article titled “Factors affecting utilization of mental health services from Primary Health Care (PHC) facilities of western hilly district of Nepal: The roles of stigma, awareness and availability”. All the comments made are heartily acknowledged. Please find the responses on the comments made on your review. The marked-up copy and an unmarked version of revised paper has also been submitted for your perusal.

**Background:**

**Comment:** Importance of conducting research in this district not clear? Why rural vs urban area was chosen?

**Response:** The comments are well acknowledged and the necessary changes are made.

The importance of conducting the research is provided from Line 105 to 124 which explains that only organizational, community and policy level factors from the socio-ecological levels had been identified in previous studies, thus this study was done to explore factors at all the levels of socio-ecological model proposed by Urie Bronfenbrenner (individual, interpersonal, organizational, community and policy level).

The rationale for conducting the research in the district are provided under “Introduction” section of the manuscript, as suggested (line 112 to 115).

The study does not compare between rural and urban areas, however participants from both the areas were selected so as to provide representation from both the areas. This is provided in detail under “Sampling procedure and sample size” topic in “Materials and Methods” section (line 165 to 179).

**Comment:** Explain what MNS mean and what disorders it represents. Dementia statistic was mentioned. Were people with dementia interviewed as well?

**Response:** The comment is well acknowledged and the explanation has been provided.

MNS disorder was termed by World Health Organization in order to incorporate mental, neurological, and substance use disorders in a single term which are often otherwise separated into treatment silos such as neurology, psychiatry, psychology, substance use, etc. in developed countries.

Reference: Altevogt BM., Hanson SL., Ssali ZN, Cuff P, Rapporteurs. Mental, Neurological, and Substance Use Disorders in Sub-Saharan Africa: Reducing the treatment gap, Improving quality of care. 2001. ISBN: 9780309148801

People with dementia were not interviewed in the study. The statistics were provided as the five most common MNS disorders include dementia. People with mental illnesses who were recovering from their illnesses were only interviewed, in presence of psychiatrist in nearby room.

**Comment:** Please make sure you are consistent on wording either use mental illnesses or mental health disorders. If you are talking about mental illnesses in general term, I would suggest using it as plural term than singular.

**Response:** The comment is well acknowledged and the necessary changes have been made. The term “mental illnesses” has been used throughout the paper, as suggested.

**Comment:** Pg. 3 line 59, mental disorders should be mental health disorder (throughout the paper, this should be changed), but I would recommend either using mental illnesses throughout or mental health disorders throughout.

**Response:** The comment is well acknowledged and the necessary changes have been made. The term “mental illnesses” has been used throughout the paper, as suggested.

**Comment:** Pg. 4 Line 75: should be mental health care instead of mental care

**Response:** The comment is well acknowledged and the necessary change has been made, as suggested.

**Comment:** Please provide some background information on mental health care provider in Nepal, in particular the district (region), where this study was conducted. Also, types of mental health disorders.

**Response:** The comment is well acknowledged and the necessary information has been provided.

The type of mental illnesses within the district have been provided in the “Introduction” section, as suggested, along with the rationale for selection of the district for study.

The health care service providers have been listed in the “Service provider” sub-topic of “Operational Definition” topic in “Materials and Methods” section (Line 147 to 149).

**Comment:** Pg 5, line 88, not sure what mhGAP resources means

**Response:** The comment is well acknowledged and the necessary information has been provided.

mhGAP operations manual, training manual and intervention guide endorsed by World Health Organization are the mhGAP resources that are available to help reduce the mental health treatment gap and to enhance the capacity of Member states to respond to the large burden of Mental, Neurological and Substance use disorders.

Reference: <https://www.who.int/mental_health/mhgap/en/>

**Comment:** Line 91: YLDs and DALYs, what do these terms mean?

**Response:** The comment is well acknowledged and the necessary changes has been made.

The full forms for the abbreviations have been provided in their first use.

YLD means Years Lived with Disability and DALY means Disability Adjusted Life Years.

**Comment:** Line 93: How is teenage childbearing related to treatment gap?

**Response:** The comment is well acknowledged.

The statement was made based on a previous study.

Reference: Kohn R, Saxena S, Levav I, Saraceno B. The treatment gap in mental health care. Bull World Health Organ. 2004;82(11):858–66.

**Comment:** Please rewrite sentence starting at line 94 With difficulties even in….to make simple and clearer

**Response:** The comment is well acknowledged and the statement has been re-written as below.

Many socio-cultural and psychosocial barriers in demand for mental health care services as well as pragmatic and health system functioning barriers along with difficulties in accessing health care has widened the treatment gap of mental illnesses at PHC facilities.

**Comment:** Line 107: Please briefly describe Bronfenbrenner model.

**Response:** The comment is well acknowledged and the description is provided as below.

The five socio-ecological levels of the model in which the study is based upon (Bronfenbrenner model) are:

i. individual level that includes characteristics that influences behavior such as knowledge, attitude and skill
ii. interpersonal level that includes social networks like partners, friends and families
iii. organizational level that includes formal and informal structures, environment and ethos
iv. community level that includes established cultural norms and values
v. policy level that includes national and local policies.

**Comment:** I would refrain from using mental ill patients, please change these phrases (through out the paper) to patient with mental illness or patient with mental health disorder.

**Response:** The comment is acknowledged and the necessary changes have been made throughout the paper, as suggested.

**Methods:**

**Comment:** Pg. 6 Operational definition, b. How did FCHVs identify patients with mental illnesses? Was this volunteer doing this for the research?

**Response:** The comment is well acknowledged and the following changes are made in the operational definition.

People with mental illnesses: Patients under psychiatric medications for mental illnesses whose identity were informed by Female Community Health Volunteers (FCHVs), PHC health workers or secondary health service providers.

**Comment:** Pg. 7, line 143: I would recommend changing to 2.6% individuals with disability rather than disabled population.

**Response:** The comment is well acknowledged and the change have been made as per suggestion.

**Comment:** Line 144-146: Does it mean that the district is not included in ongoing national mental health survey, is that one of the reasons to select this as a research site? Why is this district not included in national survey? This should be described as part of background and significance section. Also, please provide the statistics on MNS disorders in this district and region and how they differ from national data. This information should be in background.

**Response:** The comment is well acknowledged and the rationale for selection of the district as study site and the reason for the district not being included in the national survey has been provided in “Introduction” section as suggested.

Respected Reviewer, we would like to notify that Nepal does not have a national data on MNS disorders as well as statistics for the district, and thus the data could not be provided. The reason for the national mental health survey is itself to have a national statistic on mental health.

Reference: <http://nhrc.gov.np/wp-content/uploads/2019/04/Pilot-national-mental-health.pdf>

**Comment:** Please explain if the participants with mental illnesses were already patients at the PHC. How were they identified/diagnosed?

Response: The comment is well acknowledged.

The operational definition for “People with mental illnesses” is provided in line 140-143 as

Patients under psychiatric medications for mental illnesses from any health facilities whose identity were informed by Female Community Health Volunteers (FCHVs), PHC health workers or secondary health service providers.

The operational definition is to clarify that these people were patients and under treatment at any health facilities. The information regarding people under psychiatric medications were provided by Female Community Health Volunteers (FCHVs), PHC health workers or secondary health service providers.

**Comment:** Please make sure all the abbreviations are spelled out in their first use in the paper.

**Response:** The comment is well acknowledged and the necessary changes are made as per suggestion.

**Comment:** Table 1: this table can be used in supplement materials. I would rather have descriptive table in the manuscript rather than supplement. For demographic descriptive table in supplement (this should be moved to main document), I would use descriptive statistic in mean (SD), frequency (%), and/or range, rather than describing each participant. Provide descriptive statistic for each categories: mean age of participants, frequency for gender, ethnicity, religion, marital status, education, and occupation. I would provide frequency for different mental health disorders of the patients as well. It is not explained anywhere what is the mental health disorders of the participants interviewed.

**Response:** The comments are well acknowledged and the necessary changes have been made.

The descriptive statistics has not been shown in mean (SD), frequency (%) and range owing to the purposive nature of participant selection. Moreover, the socio-demographics were gathered in order to provide background characteristics of “WHO” is providing the narration rather than for any comparisons.

In addition, the interviews were mostly conducted with primary caretaker of the people with mental illnesses and thus the exact type of the mental illnesses were not collected.

**Comment:** Conceptual model should be part of the main paper as well, not supplement.

**Response:** The comments are well acknowledged and the necessary changes have been made. The result of the thematic analysis based on the theoretical model has been provided as Figure 1 in the revised paper.

**Comment:** I had hard time figuring out thematic mapping and coding document. It would certainly help if the words and figure were clearer. However, I am not a qualitative researcher, so I would rather not comment on those two documents.

**Response:** The comment is well acknowledged and the changes have been made.

Respected Reviewer, the result from the thematic analysis has been provided as Figure 1 in the revised paper.

**Comment:** Were participants compensated for their time in anyway?

**Response:** The comment is well acknowledged. As the participation was completely voluntary thus no compensation was provided for the time provided by the participants.

Modification in the statement (line 212-213) has been made.

“The decision for participation in study was voluntary without any compensation for the time provided.”

**Comment:** What does nearest caretaker mean? I think it could be substituted by primary caretaker? Is this person responsible for majority of the patient’s informal care? then I would substitute the nearest word to primary.

**Response:** The comment is well acknowledged and the necessary changes has been made, as suggested.

**Comment:** For quotes in italics, they should be indented as well.

**Response:** The comment is acknowledged and the necessary changes have been made throughout the paper, as suggested.

**Results:**

**Comment:** Pg. 12, line 237, Did you mean “misunderstood mental illnesses as communicable disease”? or “treated mental illnesses as they were communicable diseases”?

**Response:** The comment is acknowledged and the change has been made to “misunderstood mental illnesses as communicable diseases”.

**Comment:** If you are taking about mental illness as general term, I would write it as plural term: mental illnesses.

**Response:** The comment is acknowledged and the necessary changes are made throughout the paper, as suggested.

**Comment:** You should explain what “dhami/jhakri” mean for Non-Nepali readers.

**Response:** The comment is well acknowledged and the traditional/faith healers are identified as “dhami/jhakri” in line 301-302.

**Discussion:**

**Comment:** What were the difference between the study you cited? It is mentioned that there was partial similarity, what was not similar? Again, what is MNS and what disorders it encompasses should be explained in background section.

**Response:** The comment is well acknowledged. The factors identified in the previous study has also been listed and changes made in the sentence.

**Comment:** Line 483-484: “highly present among people of high economic and caste status as well as those with more education and resources”: do you mean with both high and low economic and education resources?

**Response:** The comment is well acknowledged. The study had identified that ignorance was high among people with high economic, caste and education status. The sentence in the revised paper has been modified as below:

However, a qualitative formative study done in Nepal at community and health facility level has identified that ignorance about mental health issues was highly present among people of high economic and caste status and among people with more education and resources.
