# Supplementary material for: Factors affecting utilization of mental health services from Primary Health Care (PHC) facilities of western hilly district of Nepal
Source: PLoS One. 2021 Apr 30;16(4):e0250694. doi: 10.1371/journal.pone.0250694 (PMC8087454; doi:10.1371/journal.pone.0250694)
Supplement: Attachment — Submitted filename: Response to Reviewers.docx [file pone.0250694.s013.docx]

Reviewer #1: all comments have been addressed. Data is not available but authors have provided reasons for not making them available.

**Response:** Respected Sir, the comment is well acknowledged.

Reviewer #2: The authors have addressed the issues raised by the previous reviewer/s well.
Here are some of the major concerns regarding the study.

1. Title: The authors have explored various factors affecting service utilization in PHC facilities in the district rather than "the roles of stigma, awareness and availability". The roles of stigma, awareness and availability have not been explored in detail as well. So, the mention of "the roles of stigma, awareness and availability" in the title itself is not well justified.

**Response:** Respected Sir, the comment is well acknowledged. The title has been refined as suggested.

2. How many of the primary health service providing centers in the study area had availability of mental health services? Non-availability of mental health service can be considered as a barrier to mental health service provision but definitely not a barrier to "utilization". This needs to be clarified further.

**Response:**Respected Sir, the comment is well acknowledged. The fact regarding presence of psychological counseling and referral services has been added.

3. It is the lived experiences of those with mental illness and then their caretakers that provide more authentic information regarding individual and interpersonal factors, and even community level factors that facilitate or hinder service utilization. However, the narratives of those with mental illness and that of their caretakers have been grossly under-represented in the article. Would recommend to include more of the narratives from them.

**Response:** Respected Sir, the comment is well acknowledged. Narratives from people with mental illnesses or their primary caretakers has been increased throughout the result section.

4. Conclusion- Lines 678-683 (Community awareness can be provided through mass media, campaigns, and celebrations of days related to mental health. Mental illnesses support groups can be formed to reduce stigma at the community level and increase mental health literacy at the individual level through various programs. Inclusion of mental health education in the curriculum can also help arouse awareness and mental health literacy from an early age. These support groups and inclusion of mental health education in the curriculum can help reduce and/or eliminate stigma and increase awareness in community) have not been substantiated from the results or discussion portion of the manuscript. Better to either substantiate these claims in the conclusion or drop them out.

**Response:** Respected Sir, the comment is well acknowledged. The necessary changes as suggested have been made in the conclusion section.

5. Calculation of "Inter-coder positive percent agreement"- It needs more elaboration. As per the table, all the codes from coder 2 must have matched with that of coder 1 for this gross calculation provided in the table. However, I would recommend more expert opinion in this regard.

**Response:**Respected Sir, the comment is well acknowledged. Expert opinion was provided by psychiatrist on the codes generated and the calculation of intercoder reliability. All the codes of Coder 2 did match with the codes of Coder 1. A simple percentage calculation was done then after.

Line 86- the inference of decreasing service utilization is not substantiated as the factors mentioned in the sentence refer to lack of service availability itself. So, this does not relate to "service utilization".

**Response:** Respected Sir, the comment is well acknowledged. The portion of the sentence “thereby decreasing service utilization” has been removed.

Line 97/98- "impairing family function(1), and increasing the risk of teenage childbearing(2) and domestic violence(3)"- this is cited from - 1. Keitner GI, Ryan CE, Miller IW, Kohn R, Bishop DS, Epstein NB. Role of the family in recovery and major depression. American Journal of Psychiatry 1995;152:1002-8. 2. Kessler RC, Berglund PA, Foster CL, Saunders WB, Stang PE, Walters EE. Social consequences of psychiatric disorders. II: Teenage parenthood. American Journal of Psychiatry 1997;154:1405-11. 3. Zlotnick C, Kohn R, Peterson J, Pearlstein T. Partner physical victimization in a national sample of American families: relationship to psychological functioning, psychosocial factors and gender. Journal of Interpersonal Violence 1998;13:156-65- in the article mentioned in your reference. So, while citing reference mentioned in other articles use appropriate referencing.

**Response:** Respected Sir, the comment is well acknowledged. Different studies that had identified the factors have been added as suggested.

Line 199/200- psychiatrist being available nearby in order to manage any deviation from normal seen in the patients, if required.- Please clarify what does "manage any deviation from normal seen in the patients"

**Response:** Respected Sir, the comment is well acknowledged. The sentence has been modified to provide understandable meaning.

Line 244- mentally ill patients - patients with mental illness

**Response:** Respected Sir, the comment is well acknowledged. The change as per suggestion has been made.

Two of the themes(stigma, educational level) explained in the "Mental Illness and Help Seeking pathway" do not concern directly to help seeking pathways. Also, sudden mention of "help seeking pathway" does not seem relevant to factors affecting service utilization as mentioned in the title.

**Response:** Respected Sir, the comment is well acknowledged. “Mental Illness and Help Seeking Pathway” was provided as background for the factors identified, which has been removed as suggested.

Line 402-404- It's obvious that one would not go to a facility where service is not available. So, this statement cannot be interpreted as a "barrier to service utilization". There would be no question of service utilization when service is not even available."Unavailability of service" is definitely a barrier to mental health service provision but not a barrier for "utilization". There would be no question of "service utilization" when the service does not even exist.

**Response:**Respected Sir, the comment is well acknowledged. The narration has been substituted with more relevant narrations.

The verbatim given in lines 431-432 do not substantiate the claim of "commercialization of health services" as barrier to service utilization.

**Response:** Respected Sir, the comment is well acknowledged. Presence of commercialization was acknowledged by elected representatives and the health administrators of the district.

Similarly, referral from the PHCs themselves should not be interpreted as "barrier to service utilization" at PHC. (lines 428-435)

**Response:** Respected Sir, the comment is well acknowledged. Substantial narrations have been provided.

Lines 461-468- Here, issue related to stigma have been raised. But again, there are verbatim of service provider/administrator only. It would be more relevant if the issue of stigma has been raised by the service users.

**Response:** Respected Sir, the comment is well acknowledged. Verbatim from service users has been added as suggested.

Line 518-519- "whereas some traditional healers suggested not visiting health facilities" is not substantiated by the verbatim mentioned in line 522-23 "He told me to keep my523 wife there for 7 days in order to cure her, and said that doctors would take 15 days to cure her."

**Response:** Respected Sir, the comment is well acknowledged. The necessary changes in the statement have been made.

Lines 550-560- there's national mental health policy in Nepal (<https://publichealthupdate.com/mental-health-policy-nepal/>) and policy to provide mental health services from PHC services. Local bodies do not make their own mental health policies. So, the statements basically shows the lack of awareness among the participants. It's better to clarify these issues rather than just mentioning and interpreting the verbatim of participants.

**Response:** Respected Sir, the comment is well acknowledged. Necessary changes as per suggestions have been made.

Reviewer #3: This is an important area of research, thank you for the study. The paper contains rich information on barriers to use mental health service. However, I recommend following modifications:

Prevalence of Mental Disorders in Nepal may be added in the first paragraph after the global prevalence

**Response:** Respected Madam/Sir, the comment is well acknowledged. Prevalence of Mental Disorders in Nepal has been added as suggested.

59/60: advise to reframe the sentence “This is a stark reminder of the immense burden of the huge gap in mental health”

**Response:** Respected Madam/Sir, the comment is well acknowledged. As the paragraph tried to present the burden of treatment gap in “inverted triangle” format, the sentence has been removed.

61: definition of treatment gap may not be needed; it has already been explained in lines 57-59
Treatment gap in nepal may also be added along with the treatment gap in India. The fact sheet on the finding of National MH survey of Nepal has been published.

**Response:** Respected Madam/Sir, the comment is well acknowledged. Definition of treatment gap has been removed and findings from National Mental Health Survey have been added as per suggestion.

82: what do you mean by senior staff?

**Response:** Respected Madam/Sir, the comment is well acknowledged. The term was used as such from the study cited. “Senior Community Medicine Assistant” as used in the study cited has been added.

110 – 113 aren’t these methodological issues relating to site selection?

**Response:** Respected Madam/Sir, the comment is well acknowledged. The sentence from the lines 110-113 have been kept in the “Setting” sub-heading of “Materials and Methods” as suggested. Moreover, the major mental disorder present in the diseases have also been kept within the sub-heading as it describes the status of mental disorder in the study area/site.

151: it would be useful to state whether primary care centers (health posts/PHCCs etc) in the district provide mental health services as part of government’s program or NGO program. As availability is important factor for service use and also focus of this paper.

**Response:** Respected Madam/Sir, the comment is well acknowledged. The availability of mental health services has been provided in the “Setting” sub-heading.

298-300: Suggest reviewing the write up. The line read as ‘educated people DO NOT have stigma at all’ which is not a consistent observation in researches published.

**Response:** Respected Madam/Sir, the comment is well acknowledged. The portion has been removed as it was used only to provide background in to the mental health context in the study site.

452: ‘Lack of psychiatrists, mental health experts or doctors in the PHC facilities acted as barriers for mental health service utilization’ I would suggest interpreting this as people interest to seek care from experts and not form general health care providers at PHCs. Otherwise, it suggests to place experts at PHCCs which is not the philosophy of primary care services.

**Response:** Respected Madam/Sir, the comment is well acknowledged. Changes in the sentence as per suggestions have been made.

The sections in settings (Methods) or the results did not provide information on whether the health care providers at the PHCCs are trained/supervised to provide MH service. This is relevant because without training and supervision, primary care providers generally will not be able to provide services in Nepal. Therefore, we would not be able to make inferences from statements where service users felt that PHCCs do not provide MH service. If PHCCs do not provide service for MH the problem is not in KNOWING, it is in AVAILABILITY. On the other hand, if health care providers are trained but people do not know it is different case. It would be great if authors could clarify on this.

**Response:** Respected Madam/Sir, the comment is well acknowledged. The availability of psychological counseling and referral services regardless of training and supervision provided has been added to the section.

633: suggest replacing ‘many mental health services’ to ‘basic mental health services’ … use psychotropic medications at all places

**Response:** Respected Madam/Sir, the comment is well acknowledged. The suggested change has been made.

644: do you mean inadequate implementation? I am not convinced that every municipalities should have separate ‘Policy’ for mental health. What probably was meant by research participants is MH programs or even activities at municipal level.

**Response:** Respected Madam/Sir, the comment is well acknowledged.The changes as per suggestions has been made.

678 – 685: These are the recommendations and do not seem to have come from the study participants as described in the results sections. I suggest to exclusively limit the conclusion on the finding from the study and not on the ‘opinion of the authors.

**Response:** Respected Madam/Sir, the comment is well acknowledged. The conclusion has been modified as per suggested.
